# Age-Related Interference between the Selection of Input-Output Modality Mappings and Postural Control—a Pilot Study

**DOI:** 10.3389/fpsyg.2017.00613

**Published:** 2017-04-21

**Authors:** Christine Stelzel, Gesche Schauenburg, Michael A. Rapp, Stephan Heinzel, Urs Granacher

**Affiliations:** ^1^Division of Social and Preventive Medicine, University of PotsdamPotsdam, Germany; ^2^International Psychoanalytic UniversityBerlin, Germany; ^3^Division of Training and Movement Sciences, University of PotsdamPotsdam, Germany; ^4^Clinical Psychology and Psychotherapy, Freie Universität BerlinBerlin, Germany

**Keywords:** cognitive-postural dual task, postural stability, working memory, modality compatibility, aging

## Abstract

Age-related decline in executive functions and postural control due to degenerative processes in the central nervous system have been related to increased fall-risk in old age. Many studies have shown cognitive-postural dual-task interference in old adults, but research on the role of specific executive functions in this context has just begun. In this study, we addressed the question whether postural control is impaired depending on the coordination of concurrent response-selection processes related to the compatibility of input and output modality mappings as compared to impairments related to working-memory load in the comparison of cognitive dual and single tasks. Specifically, we measured total center of pressure (CoP) displacements in healthy female participants aged 19–30 and 66–84 years while they performed different versions of a spatial one-back working memory task during semi-tandem stance on an unstable surface (i.e., balance pad) while standing on a force plate. The specific working-memory tasks comprised: (i) modality compatible single tasks (i.e., visual-manual *or* auditory-vocal tasks), (ii) modality compatible dual tasks (i.e., visual-manual *and* auditory-vocal tasks), (iii) modality incompatible single tasks (i.e., visual-vocal *or* auditory-manual tasks), and (iv) modality incompatible dual tasks (i.e., visual-vocal *and* auditory-manual tasks). In addition, participants performed the same tasks while sitting. As expected from previous research, old adults showed generally impaired performance under high working-memory load (i.e., dual vs. single one-back task). In addition, modality compatibility affected one-back performance in dual-task but not in single-task conditions with strikingly pronounced impairments in old adults. Notably, the modality incompatible dual task also resulted in a selective increase in total CoP displacements compared to the modality compatible dual task in the old but not in the young participants. These results suggest that in addition to effects of working-memory load, processes related to simultaneously overcoming special linkages between input- and output modalities interfere with postural control in old but not in young female adults. Our preliminary data provide further evidence for the involvement of cognitive control processes in postural tasks.

## Introduction

The risk of falls is significantly higher in old compared to young adults and fall-related injuries severely threaten old adults' quality of life (Tideiksaar, 1996). Adequate levels of postural control are crucial for the successful performance of activities of daily living and to avoid falls. In everyday life, however, postural tasks are rarely performed in isolation but usually combined with cognitive activities. Age-related decline in performing such combined cognitive-postural activities, i.e., the concurrent performance of a postural and a cognitive task has also been related to an increased fall-risk in old age (Bergland and Wyller, 2004; Lajoie and Gallagher, 2004; Boisgontier et al., 2013). This cognitive-motor dual-task decline in performance might be related to age-related decrements in (i) postural stability *per se* (Granacher et al., 2011), (ii) working memory capacity (Sander et al., 2012; Heinzel et al., 2014), or (iii) specific executive functions involved in coordinating concurrent task performance (Walshe et al., 2015). Here, we directly compared age-related effects on (i) cognitive and postural single tasks (ii) cognitive-postural tasks with different working-memory load (cognitive single vs. cognitive dual task), and (iii) cognitive-postural tasks requiring specific executive functions involved in the coordination of concurrent response-selection processes to different degrees.

Postural control involves controlling the body's position in space for the dual purposes of stability and orientation (Shumway-Cook and Woollacott, 2007). The alignment of posture is not just a passive state but requires the processing and integration of multiple information streams, e.g., proprioceptive, cutaneous, visual, and vestibular sensory processing (Peterka, 2002). Accordingly, postural stabilization involves the recruitment of lower level peripheral factors on the brain-stem level (Honeycutt et al., 2009) as well as higher (central) level control involving cortical and direct cortico-spinal processing (Taube et al., 2008; Taubert et al., 2010). More specifically, the regulation of posture has been related to cerebellar-cortical and fronto-striatal interactions (Jacobs and Horak, 2007; Mihara et al., 2008). In addition, functional imaging studies further support the activation of basal ganglia when imagining upright stance (Jahn et al., 2004). Studies on postural control in old age indicate that supraspinal contributions become more important as people age (Baudry, 2016), thus providing a greater potential for interference with cognitive tasks due to overlapping cortical recruitment (Herath et al., 2001). Note however, that the identification of the specific cortical sub-regions involved in postural control is still rather vague as actual postural control tasks cannot be directly performed in high-resolution functional imaging environments.

Alternatively, cognitive-postural dual-task designs can be applied to investigate, which cognitive tasks actually interfere with postural control. This, in turn, gives us direct evidence about the psychological mechanisms interfering with postural control and indirect evidence about the underlying cortical contributions to postural control.

From a psychological perspective, the dominant view holds that more attentional resources are dedicated to postural control in old age, which in turn interferes with attentionally demanding cognitive tasks (Huxhold et al., 2006; Rapp et al., 2006; Doumas et al., 2008; Berger and Bernard-Demanze, 2011; Granacher et al., 2011). In line with this, limited attentional resources have been shown to also predict falls in older individuals (Woollacott and Shumway-Cook, 2002). According to this view, more demanding postural tasks should generally lead to more interference with resource-demanding cognitive tasks in old age and vice versa (Woollacott and Shumway-Cook, 2002; Fraizer and Mitra, 2008; Boisgontier et al., 2013).

In contrast to this “limited resource hypothesis” (Kahneman, 1973; Wickens, 1980), specific cognitive control processes might affect the performance of a cognitive-postural dual task. Several dual-task models assume that executive control is crucial for the coordination of two temporally overlapping tasks (Meyer and Kieras, 1997; Logan and Gordon, 2001; Sigman and Dehaene, 2008). In fact, functional imaging studies provide converging evidence for this view by showing that lateral prefrontal activity is associated with specific aspects of dual-task coordination (D'Esposito et al., 1995; Szameitat et al., 2002; Schubert and Szameitat, 2003; Stelzel et al., 2008, 2009). The coordination demands associated with the concurrent performance of two cognitive tasks can be assumed to depend on different factors, such as the degree of structural or temporal overlap between the tasks (Sigman and Dehaene, 2008).

Here, we examined the effects of input-output modality compatibility, a factor, which has previously been shown to dramatically increase cognitive dual-task interference while keeping structural overlap at a minimum (Hazeltine et al., 2006; Stelzel et al., 2006; Stephan and Koch, 2010; Stelzel and Schubert, 2011). Modality compatibility refers to the similarity of stimulus modality and the modality of response-related sensory consequences, a principle based on ideomotor theory (Greenwald and Shulman, 1973; Prinz, 1990; Hommel et al., 2002). According to this view, preferred processing is assumed for stimulus-response-mappings with such a similarity between the stimulus modality and the sensory consequences of the response. Accordingly, special linkages are assumed for auditory-vocal and visual-manual tasks *(modality compatible)*, but not for auditory-manual and visual-vocal tasks *(modality incompatible)*. The latter tasks might require controlled translation from the stimulus information to the response to a higher degree (Kornblum et al., 1990), similar to overcoming prepotent response tendencies in the Stroop task. Empirical evidence for these additional processing demands stem from studies using temporally overlapping dual-task designs (Hazeltine et al., 2006; Stelzel et al., 2006; Stelzel and Schubert, 2011) and sequential task-switching designs (Stephan and Koch, 2010, 2011), combining either two modality compatible or two modality incompatible tasks. In the both contexts, averaging across the two component tasks eliminates effects of input- and output modality, pinpointing differences to interference of central translation processes and their coordination. These studies show strong increases in dual-task and task-switching costs in modality incompatible compared to modality compatible overlapping tasks, while single tasks do not differ depending on input-output modality compatibility. This suggests that the translation of stimulus information to a response in a non-preferred output modality is a capacity-limited process, which requires active coordination between tasks to a higher degree (Meyer and Kieras, 1997; Logan and Gordon, 2001; Sigman and Dehaene, 2008). This dual-task-specific effect of modality compatibility was further accompanied by increased dual-task-related activity in the left lateral frontal cortex (Stelzel et al., 2006), further suggesting that coordinating the response-selection processes of the two tasks becomes more demanding for modality incompatible mappings. Thus, the manipulation of input-output modality compatibility in a dual-task context provides a unique option to examine executive processes in dual-task coordination while keeping structural (input-/ output-) overlap and differences in working memory load at a minimum.

Importantly, aging has been shown to affect functions associated with anterior brain regions more than those associated with posterior regions (Brehmer et al., 2011; Grady, 2012; Heinzel et al., 2014, 2016). Consequently, deficits in cognitive-cognitive dual tasks in old adults have been interpreted as a decline in executive functions in several studies (Hein and Schubert, 2004; Clapp et al., 2011). This implies that old adults may show decrements in performing cognitive-cognitive dual tasks involving modality incompatible mappings as these are assumed to require executive functions and associated frontal brain regions to a higher degree.

Whether or not such specific dual-task coordination demands related to modality compatibility also interfere with postural stability is not known yet. We tested this by measuring center of pressure (CoP) displacements in young and old female participants aged 19–30 and 66–84 years, respectively while they performed different versions of a spatial one-back working memory task during semi-tandem stance on an unstable surface (i.e., balance pad) while standing on a force plate. Both groups also performed the same tasks while sitting.

In accordance with the limited resource hypothesis, we expected pronounced effects of working memory load (cognitive dual task vs. single task) on cognitive-postural task performance in old age. In addition, we hypothesized specific age-related effects of executive control (input-output modality compatibility) when two cognitive tasks are performed simultaneously with a postural task. Most importantly, we expected these effects to be reflected in increased total CoP displacements in the old but not the young adults.

## Materials and methods

### Participants

Eleven old women aged 66–84 years and 15 young women aged 19–30 years participated in this study. Senior participants were recruited via two health and rehabilitation sports clubs while young adults were mainly recruited through student mailing lists at the University of Potsdam, Germany. All participants were in healthy condition with no signs of neurological or psychiatric disorders, no hearing impairments, normal or corrected-to-normal vision, and no fall-incidents over the last 12 months prior to the start of this study. Furthermore, inclusion criteria for young women were suitability for measurement with magnetic resonance imaging (MRI), as they participated in a functional MRI study in a separate session. These data will be reported elsewhere. See Table 1 for demographic and neuropsychological data of the participants of the two age groups.

**Table 1:** **Demographic and neuropsychological data of young and old adults (means and standard deviations)**.

	**GROUPS**	
	**Young participants (*n* = 15)**	**Old participants (*n* = 11)**
Age *(years)*	24.8 (3.6)	72.9 (4.1)
Years of education	16.8 (3.0)	14.7 (3.7)
Trail making test—A *(seconds)*	23.3 (5.9)	32.6 (6.6)
Trail making test—B *(seconds)*	48.3 (13.4)	90.1 (26.0)
Digit symbol test *(number of correct matches in 90 s)*	68.9 (11.6)	49.4 (8.9)
Leistungspruefsystem (LPS)—subtest 3, *number of correct symbols*	31.1 (3.8)	20.9 (3.9)
Multiple choice vocabulary test (Mehrfachwortschatztest, MWT), *number of correct words*	30.9 (2.6)	33.2 (1.5)
Digit span forward	7.0 (1.1)	6.6 (1.0)
Digit span backward	5.8 (1.1)	4.8 (1.7)
Mini mental state examination (points)	Not assessed	29.2 (0.9)
Hand grip strength (kg)	28.2 (5.2)	21.9 (4.9)

This study was designed according to the Declaration of Helsinki and was approved by the local ethics committee of the University of Potsdam, Germany. Before the start of the study, participants were informed and signed written informed consent. Study participation was reimbursed monetarily with 7.5 € per test hour.

### Cognitive and postural tasks

**Figure 1:**
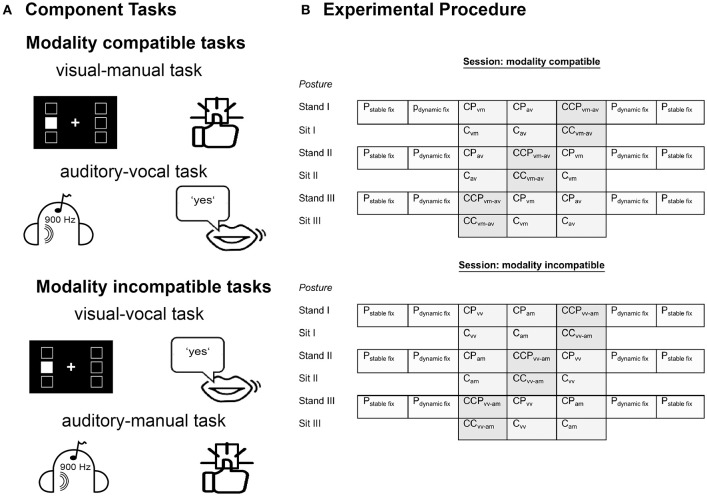
**Task design. (A)** Types of modality compatible and modality incompatible component one-back working memory tasks. Visual displays consisted of 6 possible stimulus locations, 3 to the left and 3 to the right of the fixation cross. Auditory stimuli were 3 tons of different frequencies, presented either to the left or to the right ear. Participants responded to one-back targets via button press in the manual conditions or by saying “yes” in the vocal conditions. **(B)** Study design. Each session included six runs with three standing conditions alternating with three sitting conditions. Presentation order of task blocks is shown from left to right. Each run in standing posture included seven task blocks and each run in sitting posture three task blocks each. *P*_*stablefix*_, single postural task with stable fixation; *P*_*dynamicfix*_, single postural task with dynamic fixation; vm, visual-manual task; av, auditory-vocal task; vv, visual-vocal task; am, auditory-manual task; for all other abbreviations please refer to Table 2.

Participants performed cognitive single tasks or cognitive dual tasks either with hardly any postural demands during sitting or with additional postural demands during the semi-tandem stance on an unstable surface (i.e., balance pad). In all conditions, the cognitive task included a spatial one-back task (cognitive single or cognitive dual task), which comprised either modality compatible or modality incompatible input-output modality pairings (see Figure 1A). In addition, participants also completed a postural single task without a concurrent cognitive task (visual fixation). Table 2 provides an overview of all tasks and task combinations, which will be explained in more detail below. In all tasks, participants were instructed to keep their eyes opened with the head and eyes directed toward a monitor that was individually adjusted to the respective body height of the participant. Throughout testing, participants wore headphones with an attached microphone. In addition, all participants carried a single response key in their right hand, which allowed them to press a button with their right thumb.

**Table 2:** **Overview of task conditions and abbreviations**.

	**Postural task**	**Cognitive single task**	**Cognitive dual task**
SIT	x	C (cognitive single task)	CC (cognitive-cognitive dual task)
STAND	P (postural single task)	CP (cognitive-postural dual task)	CCP (cognitive-cognitive-postural triple task)

#### Postural single tasks (P)

With their arms hanging loose to the sides of the body, participants were instructed to stand in semi-tandem stance on an unstable surface (i.e., balance pad) with the dominant leg posterior to the non-dominant leg. To determine participants' dominant leg we asked them to softly kick a ball placed approximately 1.5 m right in front of the participant. We registered the kicking leg as dominant leg. Further, participants answered two questions of the lateral preference inventory (Coren, 1993) concerning which leg they usually use when they a) want to pick something from the ground and b) should put out a cigarette on the ground. We defined the dominant leg as the leg, which was the most mentioned or used, respectively, in these three situations. The balance pad was placed on a one-dimensional force plate (Leonardo 105 Mechanograph®; Novotec Medical GmbH Pforzheim, Germany) so that total CoP were recorded during testing. Participants had to keep their head straight and their gaze fixed either on a stable visual stimulus (*stable fixation condition*) or on a dynamic visual stimulus (*dynamic fixation condition*). In the stable fixation condition, participants had to focus their gaze on a fixation cross which was presented in the center of the monitor screen. In the dynamic fixation condition, a fixation cross and an ampersand symbol (“&,” fontsize: 54) were displayed alternately in the center of the screen, with presentation times matched to presentation times in the cognitive tasks (i.e., 500 ms ampersand, 1,500 ms fixation). Here, we only report the dynamic fixation condition, as our pilot studies revealed higher postural instability during stable fixation.

#### Cognitive single tasks (C)

Participants performed different versions of a spatial one-back working memory task while sitting, i.e., with hardly any postural demands. Input stimuli were either visual or auditory and responses were given either manually or vocally. The stimulus duration was 500 ms followed by a fixation inter-stimlus interval of 1,500 ms. Task blocks consisted of 16 trials, including 5 one-back targets and 11 non-targets in pseudo-random order. According to modality compatible (i.e., visual-manual and auditory-vocal) or modality incompatible (i.e., visual-vocal and auditory-manual) input-output modality pairings, there were four different types of cognitive single tasks (see Figure 1A):

##### Modality compatible visual-manual single task

The target display consisted of a black background with a white fixation cross in the center. Visual stimuli were white squares which were presented at six different locations (up, center, down), three on each side of the fixation cross. Participants were instructed to respond fast and correct via button press when the position of the current square was the same as in the preceding trial.

##### Modality compatible auditory-vocal single task

Three different tones (200, 450, 900 Hz) were presented via headphones while a static fixation cross was displayed on the screen. The tones were presented either to the left or the right ear, resulting in 6 different stimuli. As in the visual task, participants were instructed to respond fast and correctly, when the same tone was presented to the same ear in trials n and n-1. Participants were instructed to respond vocally to target stimuli by saying “yes” (German: “Ja”).

##### Modality incompatible visual-vocal single task

The target display and stimulus presentation were the same as in the visual-manual single task, but in this case participants had to respond to target stimuli vocally by saying “yes” (German: “Ja”).

##### Modality incompatible auditory-manual single task

Targets and stimulus presentation were the same as in the auditory-vocal condition, but here participants had to respond to target stimuli manually via button press.

#### Cognitive-cognitive dual tasks (CC)

Participants performed two cognitive tasks simultaneously while sitting on a chair with a backrest. For this, a visual and an auditory stimulus were presented simultaneously for 500 ms, followed by a 1,500 ms inter-stimulus interval. Participants were instructed to decide for both stimulus modalities whether or not the stimulus was identical to the stimulus in the trial before (dual one-back task). Per task block five one-back targets were presented, i.e., two or three in the visual modality and two or three in the auditory modality. One-back targets were presented either in the auditory or in the visual modality but never simultaneously.

Both concurrent tasks were either modality compatible or modality incompatible.

##### Modality compatible dual task

Participants performed the visual-manual and the auditory-vocal task simultaneously.

##### Modality incompatible dual task

Participants performed the visual-vocal and the auditory-manual task simultaneously.

#### Cognitive-postural dual task (CP)

Participants performed the postural task (P) while simultaneously performing one of the four cognitive single tasks (C) as outlined above, i.e., either with modality compatible or modality incompatible input-output modality pairings.

#### Cognitive-cognitive-postural triple tasks (CCP)

Participants performed the postural task (P) while simultaneously performing one of the two cognitive-cognitive dual tasks (CC), i.e., either the modality compatible or the modality incompatible dual task.

### Performance assessment

#### Postural control

Postural control was assessed during semi-tandem stance (barefoot or with socks) on an unstable surface (i.e., balance pad) with the dominant leg posterior to the non-dominant leg. The balance pad (Airex®) was placed on a one dimensional force plate. Total CoP displacements (mm) were computed using CoP displacements in medio-lateral and anterior-posterior directions by means of the Pythagorean theorem. Assessment duration (33 s) was chosen in order to optimize reliability of postural stability measurement (LeClair and Riach, 1996) and in accordance with the cognitive task requirements.

#### Cognitive performance

Cognitive task stimuli were presented and manual and vocal responses were recorded via Presentation software (https://www.neurobs.com/).

### Procedure

We chose a within-subjects design and kept task and trial order the same across subjects (see Figure 1B) to allow for individual differences analyses of training effects, as this experimental protocol will be applied to examine the effects of a specific balance training in the near future.

Participants came to the biomechanics laboratory of the Division of Training and Movement Sciences, University of Potsdam for two test occasions. Test dates were separated by at least 1 week or by 4 weeks maximum. The first date comprised a neuropsychological screening procedure, including tests for vision and hearing abilities, general cognitive functioning (e.g., Mini Mental State Examination Test for seniors) and several specific neuropsychological tests (e.g., Digit Span, Trail Making A and B, see Table 1). At the end of the date, participants practiced two blocks of 32 trials for each cognitive single task and 4 blocks of 32 trials for each cognitive dual task after detailed instructions.

At the second date, participants processed the experimental tasks as outlined above while total CoP displacements and electroencephalographic (EEG) data were recorded using a mobile 64-channel EEG system. The young participants additionally participated in a functional MRI study at a third date. Further details of the neuropsychological measures, the EEG, and the fMRI data will be reported elsewhere. Here, we focus on the cognitive and CoP data, which were recorded at the second date.

The experiment during the second date consisted of two separate sessions (see Figure 1B), with six runs each. Within each run, three cognitive task blocks were performed (two cognitive single tasks, one cognitive dual task). In each session, three runs were performed in standing posture and three while sitting upright, presented in an alternating mode. Within one session, all tasks were either modality compatible or modality incompatible, respectively. The clustering of tasks into one session, which included only modality compatible tasks and into another session, which included only modality incompatible task was conducted to achieve a better level of general task performance, which might be impaired in a situation with permanent switches between these task sets. All participants performed both sessions in direct succession with a short break in-between. The test session order (modality compatible—modality incompatible vs. modality incompatible—modality compatible) was randomly assigned to participants such that half of the participants started with modality compatible tasks and half of the participants started with modality incompatible tasks.

All participants started in the semi-tandem stance condition. The standing condition always began with one stable fixation block, followed by a dynamic fixation block (33 s each to match the duration of the cognitive tasks). Thereafter, the three cognitive task trials followed (two cognitive single tasks and one cognitive-cognitive dual task, order counterbalanced across runs, 33 s each), which were again followed by one dynamic fixation block and one static fixation block. Each cognitive task block included 16 trials. While sitting, only the three cognitive task blocks were performed in the same order as in the previous standing condition.

Participants practiced the relevant tasks (modality compatible/modality incompatible) once more at this second date right before the corresponding (modality compatible/modality incompatible) experimental session in the sitting condition (one task block per cognitive single task, two task blocks per cognitive dual task).

### Statistical analyses

Performance data of the cognitive tasks were calculated as p(Hit)-p(False alarms). Vocal and manual responses were recorded during the experiment for the period of each one-back trial duration (2 s). Vocal data were analyzed offline with a self-developed Matlab tool (MathWorks; Natick, MA). The custom-made tool (Reisner and Hinrichs, 2016) was developed to facilitate automated identification of trials with correct vocal responses and to extract reaction time latencies based on simple signal amplitude measurement. The tool was validated successfully via manual coding of vocal responses (Cohens Kappa = 0.941, *p* = 0.000). Due to technical failure during recording, the vocal data of five young participants were not recorded properly and could not be analyzed. These participants were excluded from all analyses including one-back performance data. Cognitive performance data were averaged for both component tasks of each modality compatibility condition, resulting in four performance measures for the modality compatible and modality incompatible condition, respectively (C, CP, CC, CCP). These data were then subjected to a mixed general linear model, with 3 within subject factors with two levels each: 1. sit vs. stance × 2. cognitive single vs. cognitive dual task × 3. modality compatible vs. modality incompatible, and 4. age group as between subject factor. In addition to these performance measures, mean reaction times for correct target responses are reported.

As for the postural control data, we ran an exploratory data analysis using JMP® software (JMP® 8, SAS Institute GmbH, Germany) to exclude outlier blocks for each participant. Using JMP software, outlier blocks were identified by box plot analyses on the subject level and defined as blocks which were outside the whiskers, that is trials that were outside the range of < 1st quartile − 1.5^*^interquartile-range or >3rd quartile + 1.5^*^interquartile range.

Table 3 shows the average number of task blocks per condition and group included in the final data set (*n* = 15 young participants, *n* = 10 old participants). Performance data of total CoP displacements for the single postural task (P), cognitive-postural dual task (CP), and cognitive-cognitive-postural triple task (CCP) for modality compatible and incompatible mappings were calculated by averaging CoP displacements of respective conditions. Relative multiple task costs for total CoP-displacements were calculated for each run and averaged per condition (i.e., modality compatible vs. incompatible mappings) according to the formula of Doumas et al. (2008). Thus, relative dual-task costs of total CoP displacements concerning the difference between CP and P were calculated as DTCp = (CP−P)/P) ^*^ 100, whereas triple-task costs of total CoP displacements concerning the difference between CCP and P were calculated as TTCp = ([CCP−P]/P) ^*^ 100.

**Table 3:** **Means and standard errors in parentheses for number of task blocks per participant per condition and group included in analysis of total center of pressure (CoP)-displacements**.

	**GROUP**	
	**Young participants (*n* = 15)**	**Old Participants (*n* = 11)**
**COMPATIBLE SESSION**		
P-Task	5.73 (0.15)	5.82 (0.12)
CP-Task	5.50 (0.16)	5.73 (0.20)
CCP-Task	2.93 (0.07)	2.64 (0.20)
**INCOMPATIBLE SESSION**		
P-Task	5.80 (0.11)	5.91 (0.09)
CP-Task	5.87 (0.09)	6.00 (0.00)
CCP-Task	2.93 (0.07)	2.91 (0.09)

*P, Postural Single Task; CP, Cognitive-Postural Dual Task, CCP, Cognitive-Cognitive-Postural Triple Task*.

To examine assumed effects of task condition and modality compatibility, we ran a 2 (CP vs. CCP) × 2 (modality compatible vs. modality incompatible) repeated measures ANOVA with age group as between subject factor (old vs. young). For further analyses, we used planned *t*-test to elucidate which conditions drive reported significant effects. All statistical analyses were processed using IBM SPSS Statistics, Version 22.0. Effect sizes (η^2^_*p*_, d) are reported for all analyses to characterize the effectiveness of the experimental factors.

In order to directly compare trade-off effects between cognitive and postural performance, we also calculated relative dual-task costs for cognitive performance data in the cognitive single and the cognitive dual-task condition according to the formulae: DTC_CP_ = ([C−CP]/C) ^*^ 100 and DTC_CCP_ = ([CC−CCP]/CC)^*^100. These variables as well as the corresponding variables from the postural control data were then z-standardized and entered into one common repeated measures ANOVA, now including the factor performance domain (cognition vs. posture) in addition.

## Results

### Cognitive task performance

**Figure 2:**
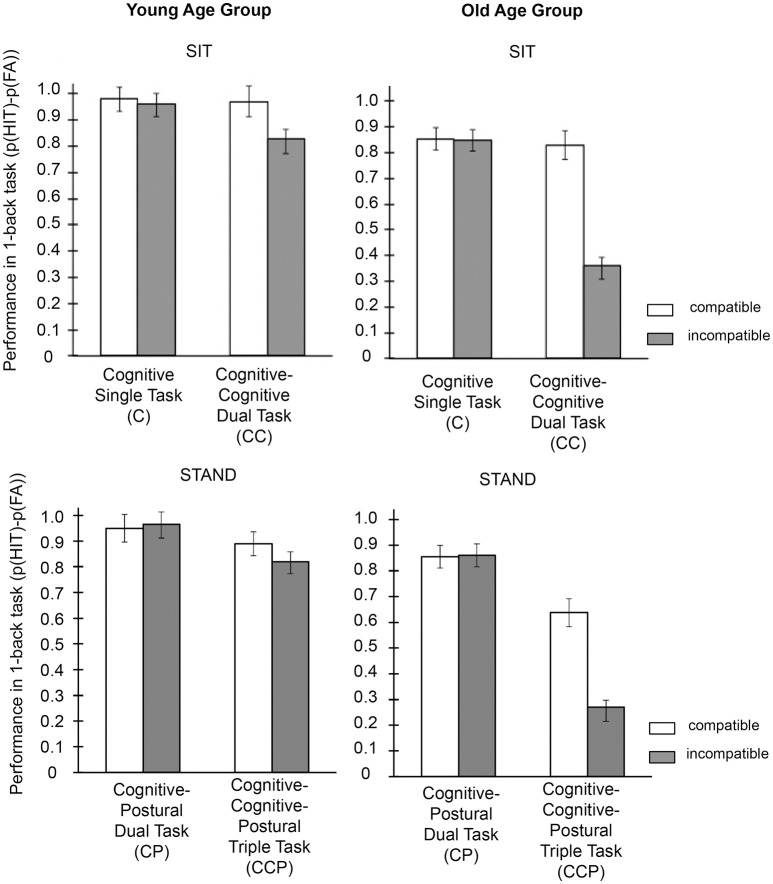
**Mean cognitive performance data defined as p(Hit) − p(False Alarm) per condition and group**.

The results of the 2 (sit vs. stance, within) × 2 (cognitive single vs. dual task, within) × 2 (modality compatible vs. modality incompatible, within) × 2 (young vs. old, between) ANOVA revealed a cognitive performance pattern consistent with (1) previous findings of selective modality compatibility effects (i.e., performance decrements for modality incompatible compared to modality compatible tasks) on cognitive dual as compared to cognitive single tasks in both age groups, (2) expected pronounced effects of working memory load (cognitive single vs. cognitive dual task) for old compared to young participants during semi-tandem stance (3) expected pronounced effects of modality compatibility for old compared to young participants during semi-tandem stance. For an overview of all cognitive performance means and reaction times per condition see Table 4 and Figure 2, for statistical results, Table 5. Note that statistical analyses are only reported for the p(Hit)−p (False Alarm) measure, which reflects performance in target and non-target trials likewise.

**Table 4:** **Cognitive performance data (p(Hit)-p(False Alarm)) and reaction times per condition (standard errors in parentheses)**.

	**SIT**		**STAND**	
	**Cognitive single task (C)**	**Cognitive dual task (CC)**	**Cognitive-postural dual task (CP)**	**Cognitive-cognitive – postural triple task (CCP)**
**p(Hit)-p(FALSE ALARM)**				
**Young Participants (*****n*** = **10)**				
Modality compatible	0.98 (0.05)	0.97 (0.04)	0.95 (0.05)	0.89 (0.05)
Modality incompatible	0.96 (0.06)	0.83 (0.03)	0.97 (0.05)	0.82 (0.04)
**Old Participants (*****n*** = **11)**				
Modality compatible	0.85 (0.04)	0.83 (0.04)	0.85 (0.05)	0.64 (0.05)
Modality incompatible	0.85 (0.05)	0.36 (0.03)	0.86 (0.05)	0.27 (0.04)
**REACTION TIMES (ms)**				
**Young Participants**				
Modality compatible	596.1 (41.7)	784.3 (56.4)	585.6 (46.0)	795.4 (45.9)
Modality incompatible	602.3 (35.6)	847.9 (56.9)	606.4 (34.9)	861.5 (69.3)
**Old Participants**				
Modality compatible	658.4 (35.5)	840.0 (48.1)	614.8 (39.2)	910.4 (39.2)
Modality incompatible	585.7 (30.3)	908.4 (48.6)	593.9 (29.8)	923.3 (59.1)

**Table 5:** **Statistical analyses of cognitive performance data (***n*** = 10 young participants, ***n*** = 11 old participants)**.

**Factor/Interaction**	***F*-value**	***p*-value**	**Partial Eta square**
**MAIN EFFECTS**			
Group	*F*_(1, 19)_ = 21.76	<0.001	0.53
Modality compatibility	*F*_(1, 19)_ = 70.86	<0.001	0.79
Cognitive Single vs. Cognitive Dual	*F*_(1, 19)_ = 49.77	<0.001	0.72
Sit vs. Stand	*F*_(1, 19)_ = 21.48	<0.001	0.53
**INTERACTIONS OF TASK FACTORS**			
Modality Compatibility × Cognitive Single vs. Cognitive Dual	*F*_(1, 19)_ = 92.21	<0.001	0.83
Modality Compatibility × Sit vs. Stand	*F*_(1, 19)_ = 8.50	0.009	0.31
Cognitive Single vs. Cognitive Dual Task × Sit vs. Stand	*F*_(1, 19)_ = 23.71	<0.001	0.56
Modality Compatibility × Cognitive Single vs. Cognitive Dual × Sit vs. Stand	*F*_(1, 19)_ = 1.96	0.177	0.09
**INTERACTIONS WITH GROUP FACTOR**			
Modality Compatibility × Group	*F*_(1, 19)_ = 24.31	<0.001	0.56
Cognitive Single vs. Cognitive Dual × Group	*F*_(1, 19)_ = 16.08	<0.001	0.46
Sit vs. Stand × Group	*F*_(1, 19)_ = 3.51	0.077	0.16
Modality Compatibility × Cognitive Single vs. Cognitive Dual × Group	*F*_(1, 19)_ = 35.46	<0.001	0.65
Modality Compatibility × Sit vs. Stand × Group	*F*_(1, 19)_ = 0.02	0.891	0.001
Cognitive Single vs. Cognitive Dual Task × Sit vs. Stand × Group	*F*_(1, 19)_ = 9.08	0.007	0.32
Modality Compatibility × Cognitive Single vs. Cognitive Dual × Sit vs. Stand × Group	*F*_(1, 19)_ = 0.65	0.43	0.03

#### Age-independent task effects

Working-memory performance in the whole group was higher for modality compatible (Mean (*M*) = 0.87; Standard Error (*SE*) = 0.03) compared to modality incompatible tasks (*M* = 0.74; *SE* = 0.03), for cognitive single tasks (*M* = 0.91; *SE* = 0.03) compared to cognitive dual tasks (*M* = 0.70; *SE* = 0.02) and for sitting (*M* = 0.83; *SE* = 0.02) compared to standing (*M* = 0.78; *SE* = 0.03). As expected, modality compatibility effects were completely triggered by the cognitive-cognitive dual-task condition (difference between cognitive dual tasks: *M* = 0.27; *SE* = 0.04) and not present in the cognitive single-task condition [difference between cognitive single tasks: *M* = 0.001; *SE* = 0.01; comparison of compatibility effects between cognitive single tasks and cognitive dual tasks, *t*_(20)_ = 6.0, *p* < 0.001, Cohen's *d* = 1.79], thus reflecting increased interference effects associated with modality compatibility. Also, the additional postural task affected modality-compatibility effects, indicating higher modality compatibility effects while sitting (*M* = 0.16; *SE* = 0.03) compared to standing [*M* = 0.11; *SE* = 0.03; comparison of compatibility effects between sitting and standing, *t*_(20)_ = 3.0, *p* = 0.007, *d* = 0.49]. This effect did not interact with the factor cognitive single vs. cognitive dual task. Finally, the effects of cognitive single task vs. cognitive dual tasks depended on the postural control condition. In other words, cognitive dual-task effects were more pronounced while standing (difference between single and dual task: *M* = 0.26; *SE* = 0.04) compared to sitting [*M* = 0.17; *SE* = 0.04, comparison of dual-task effects between sitting and standing *t*_(20)_ = 4.23, *p* < 0.001, *d* = 0.47].

#### Age-dependent effects

Working-memory performance was generally worse in old (*M* = 0.69; *SE* = 0.04) compared to young participants (*M* = 0.92; *SE* = 0.04). In addition, all main effects were more pronounced in old participants: they had stronger performance decrements in cognitive dual tasks compared to single tasks [difference between dual tasks and single tasks: old: *M* = 0.33; *SE* = 0.05; young: *M* = 0.09; *SE* = 0.02; difference between age groups: *t*_(19)_ = 4.01, *p* < 0.001, *d* = 1.75], for modality incompatible compared to modality compatible tasks [difference between modality incompatible and modality compatible tasks; old: *M* = 0.21; *SE* = 0.02; young: *M* = 0.05; *SE* = 0.02; difference between age groups: *t*_(19)_ = 4.93, *p* < 0.001, *d* = 2.15] and marginally significant higher decrements during standing compared to sitting [difference between standing and sitting: old: *M* = 0.07; *SE* = 0.02; young: *M* = 0.03; *SE* = 0.02; difference between age groups: *t*_(19)_ = 1.87, *p* = 0.077, *d* = 0.82]. Importantly with respect to aging effects on modality compatibility, the difference between modality compatibility effects in cognitive single tasks compared to cognitive dual tasks was even larger for old participants (difference in compatibility effects in dual tasks and single tasks: *M* = 0.42; *SE* = 0.05) compared to young adults (*M* = 0.10; *SE* = 0.02; difference between age groups: *t*_(19)_ = 5.96, *p* < 0.001, *d* = 2.60]. Finally, the effect of upright semi-tandem stance on decrements in cognitive-cognitive dual tasks compared to cognitive single tasks were more pronounced in old (difference in cognitive dual-task effect in standing vs. sitting; *M* = 0.14; *SE* = 0.03) compared to young adults [*M* = 0.03; *SE* = 0.02, difference between age groups: *t*_(19)_ = 3.01, *p* = 0.007, *d* = 1.32]. As the direction of the effects of upright stance on the interaction effects between modality compatibility and cognitive single task vs. dual task was the same for both groups of participants, no 4-way interaction was detected.

In sum, the cognitive performance data showed that aging affects the processing of cognitive-postural dual tasks in several ways. Besides a general performance decrement compared to young adults, cognitive-cognitive dual-task performance in old adults was severely impaired. This effect was particularly pronounced in the modality incompatible condition, which is assumed to require a high degree of executive control related to the coordination of concurrent response-selection processes. This decrement was further pronounced when old participants had to perform the postural task simultaneously, with a performance drop down to 0.27.

### Postural task performance

**Figure 3:**
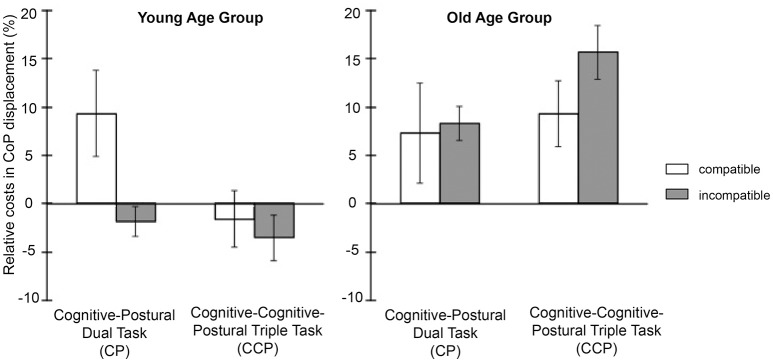
**Mean postural performance data—relative dual-task costs in total center of pressure (CoP) displacements per condition and group**.

Figure 3 illustrates the pattern of relative multiple task costs in the comparison of modality compatible and modality incompatible tasks for the young and old age group (see Table 6 for the according raw data). As can be seen from Figure 3, effects of modality compatibility on relative multiple task costs in total CoP displacements differ substantially between the young and the old age group. While the young age group showed highest CoP displacements in the modality compatible CP blocks, the old age group showed highest total CoP displacements in the modality incompatible CCP blocks, i.e., in the cognitive-cognitive-postural triple task.

**Table 6:** **Total center of pressure (CoP) displacements P-, CP-, and CCP-task per modality compatibility condition (in mm, standard error in parentheses)**.

	**TASK**		
	**Postural single task (P)**	**Cognitive-postural dual task (CP)**	**Cognitive-cognitive-postural triple task (CCP)**
**YOUNG PARTICIPANTS (*****n*** = **15)**			
Modality compatible	464.42 (24.21)	499.57 (30.10)	449.92 (20.19)
Modality incompatible	476.89 (25.37)	462.91 (19.21)	454.85 (19.73)
**OLD PARTICIPANTS (*****n*** = **11)**			
Modality compatible	763.87 (33.41)	809.58 (39.24)	807.12 (43.34)
Modality incompatible	714.82 (30.83)	769.10 (32.08)	821.49 (32.50)

A repeated measures ANOVA factoring in relative task costs for CP and CCP total CoP displacements in modality compatible and modality incompatible conditions, revealed the following results:

#### Age-independent task effects

There were no significant main effects of cognitive-postural dual task (CP) vs. cognitive-cognitive-postural triple task (CCP) and modality compatibility independent of age (all ps > 0.05), but a significant interaction of CP vs. CCP task ^*^ modality compatibility, [*F*_(1, 24)_ = 6.348, *p* = 0.019, η^2^_*p*_ = 0.209]. This interaction reflects that modality compatibility effects were generally greater in CCP task blocks (difference between modality incompatible and modality compatible: *M* = 1.5%; *SE* = 2.03) than in CP blocks [*M* = −6.02%; *SE* = 3.31; comparison of compatibility effects between CP and CCP, *t*_(25)_ = 2.69, *p* = 0.013, *d* = 0.51].

#### Age-dependent effects

Participants in the old age group had generally higher total CoP displacements during cognitive task performance, as reflected in a significant main effect of age in the analysis of the relative multiple task costs [*F*_(1, 24)_ = 8.18, *p* = 0.009, $\eta_{p}^{2}$ = 0.254]. Also, there was an interaction of CP vs. CCP task ^*^ age, *F*_(1, 24)_ = 8.763, *p* = 0.007, η^2^_*p*_ = 0.267. This effect reflects that the young age group had higher CoP displacements in CP blocks compared to CCP blocks (difference between CCP and CP: *M* = −6.27%; *SE* = 2.57), while the old age group showed a trend for the expected higher total CoP displacements in CCP blocks compared to CP blocks [*M* = 4.66%, *SE* = 2.51, difference between age groups: *t*_(24)_ = 2.96, *p* = 0.007, *d* = 1.18]. Also, there was a significant interaction effect of modality compatibility ^*^ age, *F*_(1, 24)_ = 5.344, *p* = 0.030, η^2^_*p*_ = 0.182. Numerically, young participants showed greater total CoP displacements in the modality compatible task blocks compared to the modality incompatible task blocks (difference between modality incompatible and modality compatible: *M* = −6.54%, *SE* = 3.45). In contrast, the old age group showed the expected pattern of greater total CoP displacements in modality incompatible task blocks compared to modality compatible task blocks [*M* = 3.65%, *SE* = 2.04, difference between age groups: *t*_(24)_ = 2.32, *p* = 0.007, *d* = 0.92].

Thus, while the old age group showed the expected pattern of highest total CoP displacements in modality incompatible CCP blocks, a reversed pattern was present in the young age group, with highest total CoP displacements in modality compatible CP blocks (see Figure 3). This was further supported by separate *post-hoc* independent *t*-tests on the differences in modality compatibility effects (i.e., difference between modality incompatible tasks and modality compatible tasks) in CP blocks and CCP blocks, respectively: in CP blocks the young age group had higher total CoP displacements for modality compatible tasks compared to modality incompatible tasks than the old age group [young: *M* = −11.12%; *SE* = 5.23; old: *M* = −0.1.91%; *SE* = 2.44; difference between age groups: *t*_(17.9)_ = 2.17, *p* = 0.044, *d* = 0.86], thus showing a reversed effect of modality compatibility in CP blocks in the young age group. In contrast, in CCP blocks the old age group had higher total CoP displacements than the young age group in modality incompatible tasks compared to modality compatible tasks [young: *M* = −1.91%; *SE* = 2.44; old: *M* = 6.29%; *SE* = 3.01; difference between age groups: *t*_(24)_ = 2.14, *p* = 0.043, *d* = 0.85]. Note, however, that the three way interaction of CP vs. CCP task ^*^ modality compatibility ^*^ age was not significant (*p* = 0.501), as absolute differences were in the same direction for both groups due to the negative values in the young age group.

### Integration of performance in cognition and postural control

The analysis of age-related relative dual-task costs for cognitive performance revealed generally increased relative dual-task costs for old (*M* = 8.95%; *SE* = 1.72) compared to young adults [*M* = 3.08%; *SE* = 1.72; *F*_(1, 19)_ = 5.48, *p* = 0.03, partial η^2^_*p*_ = 0.22], i.e., higher decrements of cognitive performance when standing compared to sitting in old age. In addition, old age potentiated the relative posture-related decrements in the effect of working-memory load [*F*_(1, 19)_ = 9.28, *p* = 0.007; partial η^2^_*p*_ = 0.33], i.e., cognitive dual task vs. cognitive single task performance (effect size in young: *M* = 3.57%; *SE* = 1.74; old: *M* = 18.53%; *SE* = 4.4). The ANOVA including both domains (cognition vs. posture) did not reveal any interaction with the factor domain, suggesting that no (age-related) trade-offs were present in the effectiveness of the experimental manipulations.

## Discussion

This is the first study to examine age-related interference effects between input-output modality mappings and postural control. We compared the effects of age on cognitive and postural task performance to address the question, whether aging affects (i) postural control, (ii) working memory capacity in general, and/or (iii) specific executive functions related to dual-task coordination. While there is a plethora of evidence from previous cognitive-postural dual-task studies for aging-related decrements in the domains of posture and working memory capacity, little is known about the role of specific executive functions. We hypothesized that executive functions associated with the coordination of concurrent response-selection processes related to modality compatibility (Hazeltine et al., 2006; Stelzel et al., 2006; Stephan and Koch, 2010) selectively interfered with postural control in old age. Our data provide first evidence for this assumption, showing that input-output modality compatibility has age-specific effects on both cognitive and postural performance over and above general age-related decline and effects related to increased working memory load. All age-related effects for the three domains will be summarized and discussed in the following.

### General age-related decrements

Our data replicate previous findings of cognitive performance decrements in the working-memory domain (Rajah and D'Esposito, 2005; Nyberg et al., 2012; Heinzel et al., 2014) and greater total CoP displacements during cognitive task performance (Woollacott and Shumway-Cook, 2002; Granacher et al., 2011; Boisgontier et al., 2013) in old compared to young adults. The finding of a general increase in multiple task costs for old adults support the view that independent of the specific type of cognitive task that is performed, a decline in postural stability and in cognitive information processing is present. A multitude of functional and structural changes on cortical, subcortical, and peripheral levels (Raz et al., 1997, 2005; Grady, 2012; Baudry, 2016) may account for this general performance decline in old age.

### Age-related effects of working-memory load (dual task vs. single task)

Working-memory load, as measured by differences between cognitive-cognitive dual tasks and cognitive single tasks affected cognitive task performance more in old compared to young participants, being in line with further studies on decreases in cognitive performance in old age depending on working memory load (Sander et al., 2012). It has previously been shown that old adults are able to compensate their working-memory decline to a certain degree by recruiting additional brain regions in the lateral prefrontal cortex (Reuter-Lorenz and Cappell, 2008; Barulli and Stern, 2013; Heinzel et al., 2014, 2016). This cognitive reserve, however, is limited, and the performance drop in old adults for cognitive-cognitive dual tasks in general and in cognitive-cognitive-postural triple tasks in particular suggests that increased working memory load in multiple-task situations quickly reaches this limit.

As for postural control, effects of working-memory load showed dissociable patterns for young and old adults. While old adults showed numerically higher postural instability (i.e., larger total CoP displacements) when performing cognitive dual tasks as compared to cognitive single tasks on the force plate, the reverse was true for young adults. They showed higher postural instability in the cognitive single tasks compared to the cognitive dual tasks. While the observed effects in old age are consistent with the “limited resource hypothesis” (Kahneman, 1973; Wickens, 1980), suggesting that interference arises between cognitive and postural tasks in old age because they both require limited attentional resources (Huxhold et al., 2006), the performance pattern in the young adults does not fit into that framework. Here, we expected no substantial effects of cognitive task load on postural stability, as young adults are assumed to use attentional control and supraspinal pathways to a smaller degree to control posture (Baudry, 2016). Highest instability was obtained in the easiest task condition, i.e., when modality compatible single tasks were performed. This suggests a fundamentally different processing strategy in young adults, which will be discussed in more detail further below.

### Age-related effects of executive functions (modality compatibility)

As expected, modality compatibility affected cognitive performance in both age groups only in the dual-task-context. When processing two non-preferred input-output modality mappings (i.e., visual-vocal and auditory-manual) simultaneously, cognitive dual-task performance was severely impaired compared to modality compatible mappings (i.e., visual-manual and auditory-vocal). This effect was even more pronounced in the old age group and when performing the postural control task in addition (i.e., cognitive-cognitive-postural triple task). This finding extends previous studies on the effects of input-output modality compatibility in several ways. First, it shows that modality compatibility is effective in different task settings. Previous studies used simple choice-reaction tasks in dual-task (Hazeltine et al., 2006; Stelzel et al., 2006; Stelzel and Schubert, 2011) and task-switching contexts (Stephan and Koch, 2010, 2011). Here, a one-back working memory task was applied, which did not require responses on every trial but only for one-back targets. Still, effects of modality compatibility were robust in both age groups and highly consistent with the findings in choice-reaction tasks. This suggests that the process of simultaneously keeping track of two modality incompatible task sets with the requirement to emit a modality incompatible response occasionally is highly similar to applying the mappings on every trial. This further supports the close coupling of stimulus and response information in a given task set (Greenwald and Shulman, 1973; Prinz, 1990; Hommel et al., 2002), including the idea that response information is activated even when the response is not executed. In addition, the present study is the first to show age-related decrements in the processing of modality incompatible dual tasks. This coincides with the assumption that the concurrent processing of two modality incompatible tasks is associated with increased demands in controlled dual-task coordination, which has been associated with the lateral frontal cortex (Szameitat et al., 2002; Schubert and Szameitat, 2003; Stelzel et al., 2006, 2008, 2009), i.e., the part of the brain, which shows most robust decrements in old age (Grady, 2012).

Concerning the effects of modality compatibility on postural control—CoP data in the old age group were all in the same direction as the effects in the cognitive performance data with selective increases in total CoP displacements in the modality incompatible dual task. Thus, for old adults the increased cognitive demands associated with the coordination of two non-preferred input-output modality mappings directly interfered with postural control. Note that the modality compatible and the modality incompatible cognitive dual tasks did not differ in terms of working-memory load, neither did the dual task involve overlap in perceptual or response requirements. Both dual tasks involved the simultaneous perception of a visual and an auditory stimulus and an equal number of manual and vocal responses. Furthermore, central code overlap (i.e., spatial codes in both tasks) was the same for modality compatible and modality incompatible dual tasks. Accordingly, the increased total CoP displacements cannot be associated with either of these factors, but must be related to other differences in central processing requirements. Consequently, we interpret the performance decrements with decrements in higher-order control processes associated with coordinating the concurrent translation of non-preferred input-output modality mappings that have been associated with activity in the lateral frontal cortex before (Stelzel et al., 2006).

That the recruitment of these frontal regions for the cognitive dual task interferes with postural control in old age is in line with age-related neuronal changes in this group. Age-related decrements in postural control has been described previously in the form of narrative reviews (Granacher, 2011; Granacher et al., 2012; Baudry, 2016) and original work (Lajoie and Gallagher, 2004; Berger and Bernard-Demanze, 2011; Granacher et al., 2011). With reference to these findings, we postulate that age-related changes in postural control are most likely caused by age-related changes in the peripheral and the central nervous system. In other words, numerous degenerative processes within the central nervous system (e.g., desensitization of mechanoreceptors, reduction number of sensory and motor neurons, reduced volume of gray and white matter in different brain areas etc.) are responsible for age-related performance decrements in postural control. Due to the complex interactions of the different structures within the postural control system and how these are affected by biological aging and physical inactivity, it is highly speculative and most likely inadequate to reduce age-related decrements in postural control to selected structures within the central nervous system.

Nevertheless, some work has been done, in an attempt to examine supraspinal mechanisms responsible for age-related changes (Jacobs and Horak, 2007; Mihara et al., 2008; Rosano et al., 2008; Baudry, 2016). For example, Rosano et al. (2008) assessed gray matter volume of five different brain regions and spatiotemporal gait parameters in older adults. Shorter steps and longer double support times were associated with smaller sensorimotor regions within the motor, visuospatial, and cognitive speed domains. These findings suggest that measures of gait in older adults living in the community are not only the consequence of underlying age-related changes in peripheral systems (i.e., neuropathology; Marchetti and Whitney, 2005), but that they also indicate underlying focal, selective changes in brain structure.

Further evidence for potential mechanisms underlying age-related decrements in postural control comes from studies with patients examining age-related pathologies (i.e., dementia and M. Parkinson) and their impact on postural control. Mild cognitive impairment (MCI) is often associated with changes in volume of the prefrontal cortex. Furthermore, there is evidence that MCI patients' postural control is particularly affected under dual-task conditions as opposed to age-matched healthy seniors (Montero-Odasso et al., 2012; Muir et al., 2012). In other words, it can be postulated that changes in the prefrontal cortex are associated with decrements in postural control (Sheridan and Hausdorff, 2007; Mihara et al., 2008). Moreover, Parkinson's disease is characterized by a loss of dopaminergic neurons and associated with severe decrements in postural control (e.g., freezing of gait, ataxia; Kaasinen and Rinne, 2002). Therefore, age-related changes in striato-frontal pathways appear to be directly related to postural instability.

Whether the locus of age-related changes underlying the reported decrements in the present study is the prefrontal cortex *per se* or other regions connected to the prefrontal cortex (Frank et al., 2001; Dahlin et al., 2008; Backman et al., 2011) cannot be separated in our behavioral study. Still, the present task design provides the possibility to pinpoint cognitive-postural interference effects to specific cognitive aspects relevant to dual-task processing (Meyer and Kieras, 1997; Logan and Gordon, 2001) over and above increased working-memory load. Further studies are required to more directly examine the role of executive coordinative processes in cognitive-postural dual-task situations.

Postural stability data in the young age group did not coincide with the effects of modality compatibility on cognitive performance. Increased cognitive task demands in modality incompatible dual tasks did not lead to increased total CoP displacements compared to the single motor tasks, i.e., no relative triple-task costs emerged. Instead, young participants showed greater postural sway in the seemingly easiest tasks, the modality compatible single tasks. This reversed effect in the young age group, i.e., increased postural stability and diminished multiple task cost for the most demanding task finds support by other studies reporting improved postural stability in several postural-cognitive dual-task settings (Andersson et al., 2002; Riley et al., 2003; Brauer et al., 2004; Lacour et al., 2008). This was explained by attentional effects, i.e., a change in focus regarding internal vs. external focus of attention with respect to posture depending on the task demands (Wulf and Prinz, 2001). It is assumed that as the attentional focus shifts from postural control to the cognitive task, balance will be controlled by more automatic and more efficient processes (Vuillerme and Nafati, 2007). Improvement in measures of postural control was shown in studies where the focus of attention was explicitly manipulated showing reduced body sway with an external focus of attention as compared to an internal focus of attention (McNevin and Wulf, 2002; Wulf et al., 2004). Differences in such shifts in attentional focus depending on the cognitive task requirements could provide one explanation for differential age effects. The reverse pattern of total CoP displacements in the young compared to the old age group might be further explained by an underlying inverted U-shaped non-linear interaction model (Lacour et al., 2008), i.e., for young participants task demands might have been optimal in all but the modality compatible single-task condition and therefore did not interfere with postural control. In contrast, in the old age group, already the seemingly easy modality compatible single tasks provided a challenge, which peaked in a cognitive-postural performance break down in the modality incompatible dual task. Direct manipulations of attentional focus in studies on specific executive functions in cognitive-postural dual tasks might shed further light on these mechanisms.

## Conclusion

In sum, our findings provide further evidence for age-related decrements in the concurrent performance of cognitive and postural tasks. They extend previous findings by separating effects of unspecific resource limitations from specific changes in coordinating temporally overlapping task requirements. This specification of age-related decrements provides new opportunities for cognitive-postural dual-task training procedures, which should also focus on such coordinative skills. Due to the small sample size and the inclusion of female subjects only, our findings cannot be generalized to other populations and need to be interpreted with care because they are preliminary. Future studies should replicate our approach by including larger samples and males as well as females in their cohort. Also, larger samples will allow testing for the association of cognitive-postural interference with further neuropsychological measures, which would allow a more elaborate interpretation with respect to the underlying cognitive mechanisms. Still, the robustness of effects even in this small sample of rather healthy old female adults indicate the relevance of training procedures in old adults with the overall goal of reducing fall-risk and associated decreased quality of life.

## Ethics statement

This study was carried out in accordance with the recommendations of the ethics committee of the University of Potsdam with written informed consent from all subjects. All subjects gave written informed consent in accordance with the Declaration of Helsinki. The protocol was approved by the ethics committee of the University of Potsdam.

## Author contributions

All authors listed have made substantial, direct and intellectual contribution to the work, and approved it for publication. CS and GS contributed equally as first authors. SH and UG contributed equally as senior authors.

### Conflict of interest statement

The authors declare that the research was conducted in the absence of any commercial or financial relationships that could be construed as a potential conflict of interest.
